# 38.4 PREVALENCE OF ANTI-NEURONAL ANTIBODIES IN PATIENTS ADMITTED WITH FIRST EPISODE OF PSYCHOSIS AND THEIR CLINICAL OUTCOMES

**DOI:** 10.1093/schbul/sby014.157

**Published:** 2018-04-01

**Authors:** James Scott, David Gillis, Alex Ryan, Hethal Hargovan, Stefan Blum

**Affiliations:** 1 The University of Queensland; 2 Royal Brisbane and Women’s Hospital

## Abstract

**Background:**

Anti-neuronal antibodies are associated with psychosis although their clinical significance in first episode of psychosis (FEP) is undetermined. This study examined the prevalence of anti-neuronal antibodies in patients admitted to hospital for treatment of their first episode of psychosis and described clinical presentations and treatment outcomes of those who were antibody positive.

**Methods:**

Between July 2013 and May 2015, all consenting patients aged between 12 and 50 admitted for their first episode of psychosis to three mental health hospitals in Queensland, Australia, were tested for anti-neuronal antibodies in serum. Antibody positive patients were referred for neurological and immunological consultation and treatment.

**Results:**

During the study, 154 FEP patients were admitted with their first episode of psychosis and 113 consented to participate. Six patients were found to have anti-neuronal antibodies; (anti-NMDAR antibodies [n = 4], VGKC antibody [n = 1], antibody against uncharacterised antigen [n = 1]). Of these, five received immunotherapy, leading to complete resolution of psychosis in four.

**Discussion:**

A small, but significant subgroup of patients with first episode psychosis have anti-neuronal antibodies detectable in serum and evidence of central nervous system autoimmune pathology. Early identification of these patients and referral for appropriate treatment is critical to optimise recovery.

